# Synthesis and foaming of a novel type of porous geopolymer material *via* salt activation

**DOI:** 10.1039/d5ra05707h

**Published:** 2025-10-21

**Authors:** Goryunova Kristina, Gahramanli Yunis

**Affiliations:** a Azerbaijan State Oil and Industry University Baku city Azerbaijan kristina.qoryunova.i@asoiu.edu.az

## Abstract

The construction industry's dependency on Portland cement and its poor recycling of construction and demolition waste (CDW) significantly contribute to global CO_2_ emissions and environmental degradation. This study presents the synthesis and characterization of an innovative, foamed geopolymer material derived from ceramic waste (waste bricks), activated with sodium dihydrogen phosphate and foamed using calcium carbonate and citric acid. The research aims to develop sustainable thermal insulation materials through salt activation, an underexplored alternative to traditional alkali activation. The effects of varying foaming agent content and foaming activator concentration on density, porosity, thermal conductivity, and mechanical strength were systematically investigated. The most suitable parameters yielded a bulk density as low as 525 kg m^−3^ and thermal conductivity of 0.00998 W (m K)^−1^, placing the material among the most efficient thermal insulators. True porosity reached up to 68.3%, while compressive strength ranged from 0.4 to 9.37 MPa. Microstructural analysis confirmed a hybrid aluminosilicate-phosphate network with tunable pore morphology. These results demonstrate that salt-activated geopolymers can serve as eco-friendly, low-carbon materials or have use in insulation applications, offering a viable approach to both waste valorization and CO_2_ mitigation in construction.

## Abstract 2

The research aims to develop sustainable thermal insulation materials through salt activation, an underexplored alternative to traditional alkali activation.

## Graphical abstract

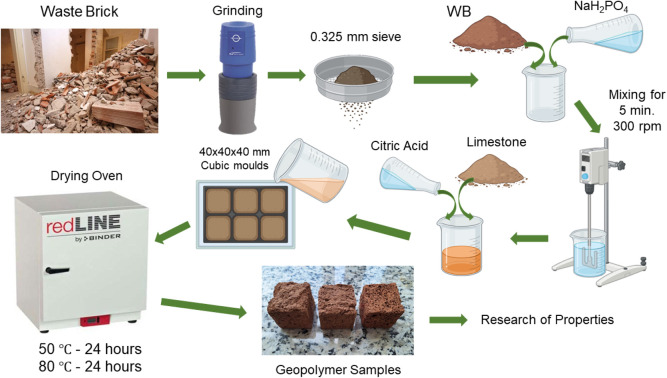

## 1. Introduction

The construction sector is one of the largest consumers of raw materials and a major source of environmental pollution. Ongoing urbanization and industrial expansion have greatly increased the demand for building materials, leading to intensified use of natural resources and elevated industrial waste production.

Despite growing awareness, the construction industry continues to rely heavily on Portland cement, a material responsible for 7–8% of global anthropogenic CO_2_ emissions.^1^ Producing one ton of cement typically requires 1.5 tons of raw materials, consumes 3.2–6.3 GJ of energy, and emits about one ton of CO_2_.^2,3^ Projections show that cement production will rise by 50% by 2050,^4^ threatening efforts to limit global warming to 1.5 °C. In response, the World Resources Institute aims to reduce CO_2_ emissions in the construction sector from 615 kg CO_2_ per t to 360–380 kg CO_2_ per t by 2030, and eventually to 55–90 kg CO_2_ per t by 2050.^5^ However, the average emission trends from 2012 to 2017 suggest these targets are unlikely to be met.

In light of these challenges, the development of environmentally friendly alternatives to cement is urgent. Another key issue is construction and demolition waste (CDW), which constitutes about 40% of global waste.^6^ This contributes to landfill overflow, soil pollution, and increased emissions. To mitigate this, the EU's Waste Framework Directive (2008/98/EC) prioritizes CDW reduction, recycling, and recovery,^7^ aiming for 70% CDW recycling by 2050.^8^ Though some countries like the Netherlands and Denmark claim success,^9^ most CDW is downcycled into low-value materials,^10,11^ revealing the need for more effective upcycling methods.

Thus, two main environmental concerns drive innovation in construction materials: the carbon footprint of Portland cement and the poor recycling of CDW. Researchers are developing materials that simultaneously replace conventional cement and integrate recycled CDW – among them, geopolymers.

The term “geopolymer” was introduced in the 1970s by French scientist Joseph Davidovits to describe aluminosilicate-based inorganic polymers formed by reacting aluminosilicate powders with alkaline solutions.^12–14^ Initially developed for fire-resistant applications, geopolymers have gained traction in the construction sector. They are primarily divided into alkaline-activated and phosphate-activated types,^15^ with the former receiving more research attention.

As global energy demands increase, so does the need for energy-efficient buildings. The construction sector alone accounts for 35–40% of total energy consumption^16^ and is a major emitter of greenhouse gases.^17^ Effective thermal insulation is key to improving building energy efficiency, reducing heating, ventilation, and air conditioning (HVAC) use, and lowering emissions. Besides energy savings, insulation materials offer additional benefits such as noise reduction and fire resistance,^18^ and they are widely used in storage and pipeline systems.^19,20^

Insulation materials are typically inorganic, organic, hybrid, or advanced, and come in various forms including porous and rigid structures.^21^ Inorganic types like glass wool and rock wool dominate the market, while polymers such as polyurethane (PU), polyimide (PI), extruded polystyrene (XPS), and expanded polystyrene (EPS) are also common due to their low thermal conductivity and cost-effectiveness.^22^

Improving the thermal insulation of inorganic materials often involves adding pores to reduce heat transfer. Foaming agents like aluminium powder, hydrogen peroxide, and sodium salts are commonly used for this purpose.^23–26^

Recent research highlights the potential of porous geopolymers as efficient, eco-friendly insulation alternatives to Portland cement-based materials.^27–29^ These are synthesized at ambient temperatures from low-cost raw materials and industrial waste, minimizing energy use and emissions. For example, in acid-activated geopolymer production, CaCO_3_ (limestone) reacts with phosphoric acid to release CO_2_, forming pores.^30^ This allows recycling of industrial by-products like phosphogypsum and phosphate washing sludge – wastes that otherwise burden the environment and limit phosphate industry growth.^31–34^

However, the use of salt activation (*via* alkali metal phosphates and dihydrophosphates) in geopolymer production has been scarcely explored. This study investigates the synthesis of salt-activated foamed geopolymers as a sustainable solution for both cement substitution and CDW utilization, aiming to reduce equipment corrosion and side reactions compared to alkali and acid activation, due to neutral environment of sodium dihydrophosphate solution. The aim of this work was to obtain geopolymer materials with a predefined porous structure for subsequent use as thermal insulation materials.

## 2. Materials and methods

### 2.1. Materials

**Fig. 1:**
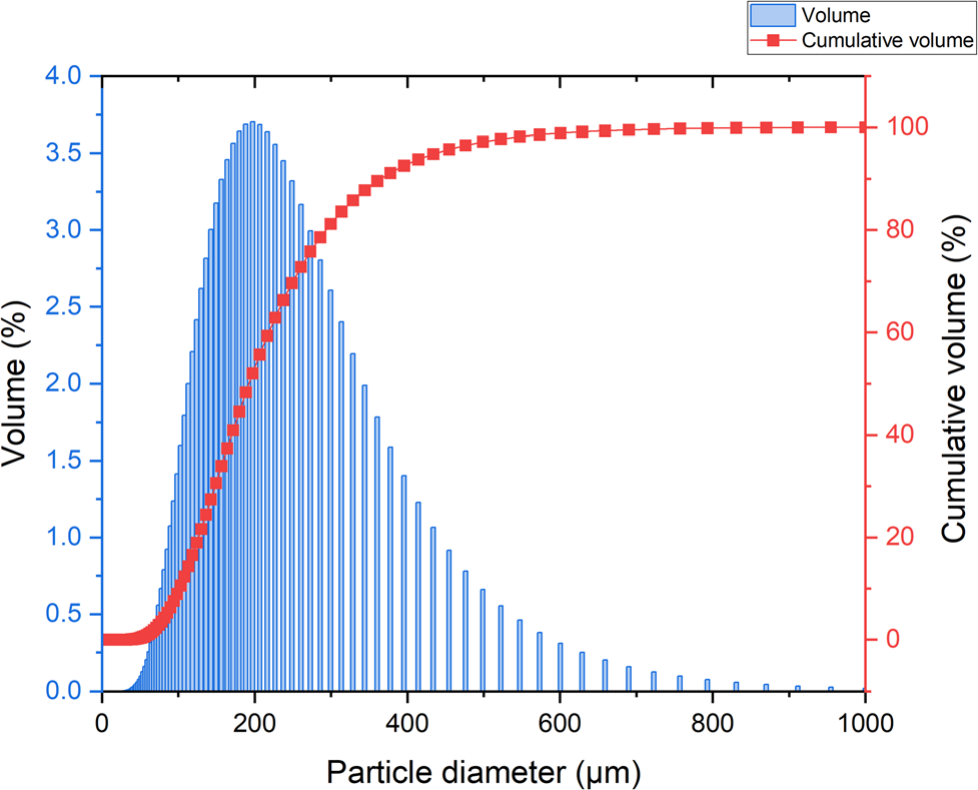
Particle size distribution of waste brick.

For the preparation of geopolymer materials we have used waste brick (WB) as raw material obtained from brick manufacturing company “Fakhraddin-K MMC” as non-conditioning product. The composition of WB provided in the Table 1. Fig. 1 presents the particle size distribution of WB, which was analysed by a laser particle size analyser Mastersizer Hydro (Malvern Instruments) at the Institute of Polymer Materials of the Republic of Azerbaijan. The median particle size of WB was about 325 μm.

**Table 1:** Chemical composition of waste brick

Na_2_O	MgO	Al_2_O_3_	SiO_2_	CaO	Fe_2_O_3_	MnO	Cr_2_O_3_	K_2_O	LOI
1.88	2.44	15.6	52.67	8.47	11.78	0.21	2.06	2.75	0.15

In the role of activator, the sodium dihydrogen phosphate (NaH_2_PO_4_) was used. As the foaming agent, the waste limestone powder (CaCO_3_), obtained from The Second Geological District of Garadakh Deposit was used. The composition of limestone is provided in the Table 2. As the activator of the foaming, the citric acid (C_6_H_8_O_7_) was used.

**Table 2:** Chemical composition of limestone

Na_2_O	MgO	Al_2_O_3_	SiO_2_	CaO	Fe_2_O_3_	K_2_O	P_2_O_5_	MnO	LOI
0.19	0.58	0.84	3.87	53.31	1.11	0.15	0.05	0.04	39.16

### 2.2. Synthesis and foaming of geopolymers

As described above, the synthesis of novel foamed geopolymer materials was carried out using waste brick (WB) as the precursor and a sodium dihydrogen phosphate NaH_2_PO_4_ (Ac) solution as the geopolymerization activator. The mass ratio of precursor was maintained at 50 : 50, with the concentration of the salt-based activating solution fixed at 60%. To generate the porous structure, calcium carbonate (CaCO_3_) served as the foaming agent, while citric acid (C_6_H_8_O_7_) was applied as a foaming activator in concentrations ranging from 10% to 50%. The amount of limestone used varied between 0.4 and 2 wt%, while the citric acid was consistently used at 2 wt% of the total mixture.

**Fig. 2:**
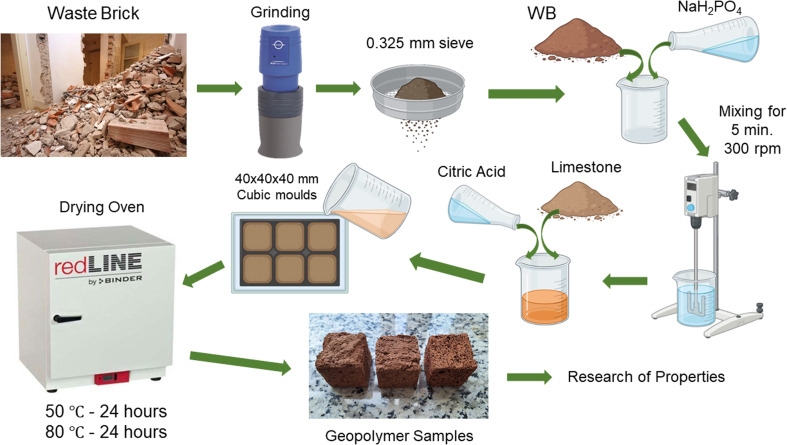
The graphical abstract of conducted experiments.

**Fig. 3:**
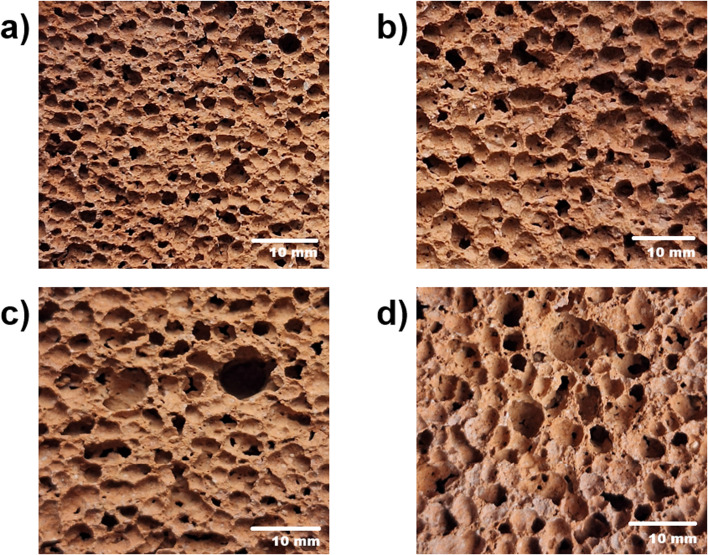
. The surface appearance of obtained porous geopolymers depending on the content of foaming agent: (a) 0.4 wt%; (b) 1 wt%; (c) 1.6 wt%; (d) 2 wt%.

The synthesis process was carried out as follows (Fig. 2): WB was grinded in IKA A11 laboratory grinding mill for 1 min at speed 28 000 rpm. Then, obtained WB powder and Ac were combined and mixed thoroughly for 5 minutes. The Table 3 represents experimental mix proportion details. Extended mixing was necessary because ceramic waste typically contains calcite, which reacts with the phosphate activator to release CO_2_. This uncontrolled gas release could result in premature foaming, so prolonged mixing helped to remove the excess gas and stabilize the mix before intentional foaming. After stabilization, a predetermined amount of citric acid solution (at varying concentrations) and limestone (in varying proportions) were introduced into the inert mixture. The blend was stirred gently to ensure even distribution of the gas-producing agents throughout the system. The resulting paste was poured into silicone moulds and left at ambient conditions overnight to allow initial hardening. Following this, the samples were subjected to a two-stage heat treatment: first at 50 °C for 24 hours, and then at 80 °C for 24 hours. Curing at ambient temperature had been carried out for 7, 14 and 28 days. After the first stage of curing, the solidified geopolymer blocks were removed from the moulds. The Fig. 3 represents the view of the porous structure of obtained samples. Obtained samples then were sent for further testing of bulk density, true, open and closed porosities, thermal conductivity, and compressive strength.

**Table 3:** Experimental mix proportion data

WB, wt%	Ac, wt%	Ac conc., %	CaCO_3_, wt%	C_6_H_8_O_7_, wt%	C_6_H_8_O_7_ conc., %	*T*, °C	Stirring speed, rpm	Stirring time, min
50	50	60	2	1	10	25	300	5
50	50	60	1.6	1	10	25	300	5
50	50	60	1	1	10	25	300	5
50	50	60	0.4	1	10	25	300	5
50	50	60	2	1	20	25	300	5
50	50	60	1.6	1	20	25	300	5
50	50	60	1	1	20	25	300	5
50	50	60	0.4	1	20	25	300	5
50	50	60	2	1	30	25	300	5
50	50	60	1.6	1	30	25	300	5
50	50	60	1	1	30	25	300	5
50	50	60	0.4	1	30	25	300	5
50	50	60	2	1	40	25	300	5
50	50	60	1.6	1	40	25	300	5
50	50	60	1	1	40	25	300	5
50	50	60	0.4	1	40	25	300	5
50	50	60	2	1	50	25	300	5
50	50	60	1.6	1	50	25	300	5
50	50	60	1	1	50	25	300	5
50	50	60	0.4	1	50	25	300	5

### 2.3. Determination of bulk density of foamed geopolymers

The volume weight of foamed geopolymer was determined according to ISO 5016:1997. The determination was carried out as follows. After removing the samples from the mould, they were additionally subjected to heat treatment at 80 °C for 16 hours. Then the samples were conditioned at room temperature for a day. After that, the main dimensions of the samples were measured using a calliper. The measurement was carried out with an accuracy of 0.01 mm. Taking into account that the moulds for the preparation of samples had the form of truncated cone, the measurement was reduced to the determination of the height of the conical sample and its large and small diameter. Measurement of the dimensions was carried out in three points and then the arithmetic mean of the measurement was calculated. Next, we had to determine the geometric volume of the foam geopolymer samples based on the measurements. Taking into account that the samples had the shape of a truncated cone, the calculation of their geometric volume was carried out according to the following formula:1
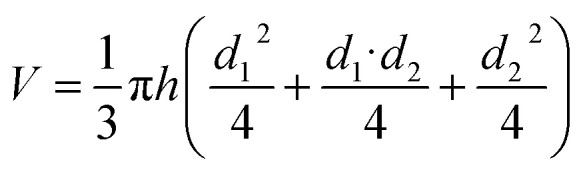
where *h* – height of the specimen, m; *d*_1_ – diameter of the lower part of the specimen, m; *d*_2_ – diameter of the upper part of the specimen, m.

The mass of the samples was also determined on analytical scales with an accuracy of 0.001 g. The bulk density of the samples or apparent density of the porous geopolymer samples was then determined using the following formula:2
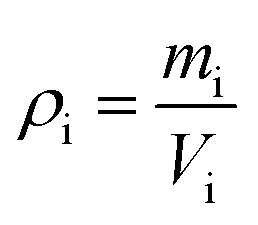
where *m*_i_ – mass of the i^th^ sample, kg; *V*_i_ – geometric volume of the i^th^ sample, m^3^.

### 2.4. Evaluation of true, open, and closed porosity

The characterization of porous materials involves the determination of true porosity, open porosity, and closed porosity. These parameters describe different aspects of the internal porous structure of a material and are crucial for understanding its performance in various applications. True porosity refers to the total volume of pores within a material relative to its overall volume, including both open and closed pores. It reflects the complete pore structure without distinguishing between interconnected and isolated pores. The true porosity of foamed geopolymer was determined according to ISO 5016:1997. The determination was carried out as follows. After determining the bulk density of foamed and monolith samples, true porosity can be calculated according to the following formula:3
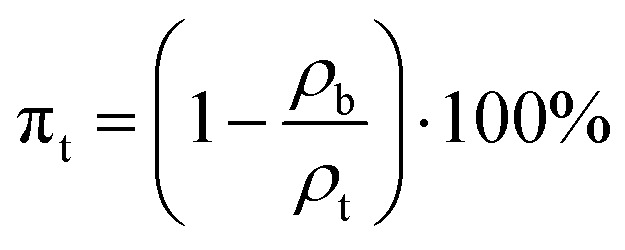
where *ρ*_b_ – the bulk density of foamed sample, kg m^−3^; *ρ*_t_ – the true density of monolith sample, kg m^−3^.

Open porosity represents the fragment of the material's volume that consists of interconnected pores accessible from the surface. These pores contribute to fluid adsorption and transport properties. The open porosity of foamed geopolymers can be determined by immersing samples into water, and can be calculated according to the following formula:4
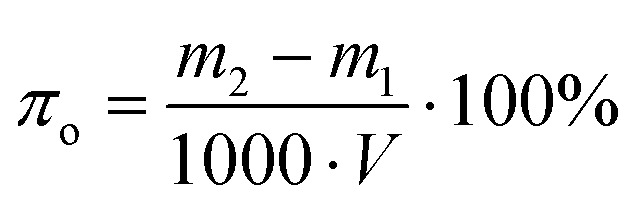
where *m*_1_ – mass of specimen before saturation, g; *m*_2_ – mass of specimen after saturation, g; *V* – the geometric volume of the sample, mm^3^.

Closed porosity describes the fraction of pores that are isolated from the external surface. These pores are enclosed within the material and cannot be penetrated by fluids. The closed porosity of samples can be determined from the difference between open and true porosity, and can be calculated according to the following formula:

5 π_c_ = π_t_ − π_o_

where π_t_ – true porosity of specimen, %; π_o_ – open porosity of specimen, %.

### 2.5. Measurement of thermal conductivity *via* steady-state technique

The thermal conductivity of the tested samples was determined in accordance with ASTM E1225-20, which specifies a steady-state technique for measuring the thermal conductivity of homogeneous opaque solids using the guarded-comparative-longitudinal heat flow method. The experiments were conducted under controlled laboratory conditions on Thermtest MP-V apparatus. Prior to each test, the entire apparatus was thermostated to ensure uniform thermal conditions throughout the system. Thermal conductivity measurements were performed at three different temperatures: 50, 70, and 90 °C. After setting the desired temperature, the system was allowed to stabilize for 30 minutes to establish a steady-state regime, during which the temperature readings remained constant. The samples used in the experiment were cylinder-shaped, with dimensions of 50 × 100 mm, as described by the standard of apparatus. Before testing, the specimens were dried at a temperature of 105–110 °C until a constant mass was achieved to eliminate moisture that could affect the measurement results. During the test, the sample was placed on the heat-measuring plate of instrument and compressed from above to ensure firm contact. Thermocouples installed on the bottom heating plate and on the upper surface of the sample recorded the temperature difference across the specimen. The values were displayed on the monitoring device and recorded once a stable reading was observed.

The heat flux is calculated using the formula:6
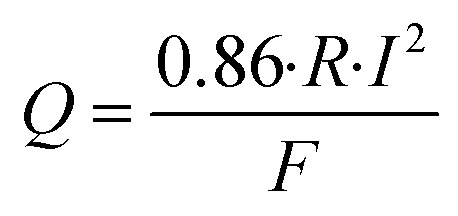
where *R* – the resistance of the heating element, ohm; *I* – the current in the circuit, A; *F* – the surface area of the heat-measuring plate, m^2^.

Using the values of the heat flux and the temperature difference between the top and bottom surfaces of the sample, the thermal conductivity coefficient is calculated with the following formula:7
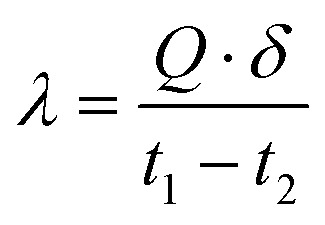
where *λ* – the thermal conductivity, W (m K)^−1^, *Q* – heat flux, kcal (m^2^ h)^−1^, *δ* – the thickness of the sample, m; *t*_1_ − *t*_2_ – the temperature difference across the sample, °C.

### 2.6. Compressive strength testing procedure

Determination of the physical–mechanical properties of the taken samples was carried out in accordance with EN 12390-3. The determination of compressive strength was performed using the “YAW-300D” testing machine with a maximum load capacity of 300 kN. During compression, the loading rate of the sample was 0.6 ± 0.2 MPa s^−1^. The foamed geopolymer samples were shaped as 40 × 40 × 40 mm cubic specimens for determining compressive strength. The kinetic deformation curves were constructed based on the graphical dependencies recorded by the testing press.

### 2.7. Fourier-transform infrared spectroscopy (FTIR) analysis

To identify the functional groups, present in the geopolymer samples, Fourier-transform infrared spectroscopy (FTIR) was performed using a PerkinElmer Spectrum Two spectrometer equipped with an ATR (Attenuated Total Reflectance) module. Prior to analysis, the samples were finely ground using a IKA A11 analytical mill to obtain a uniform powder. Approximately 5 mg of powder was placed directly onto the ATR crystal without the need for pellet formation or additional reagents. Spectra were recorded in the range of 4000–400 cm^−1^ with a resolution of 4 cm^−1^, and 32 scans were accumulated per sample to improve the signal-to-noise ratio. Background correction and calibration were performed before each measurement session. The resulting spectra were processed using the OriginLab software to identify characteristic adsorption bands associated with various functional groups.

### 2.8. X-ray diffraction (XRD) analysis

X-ray diffraction (XRD) analysis was conducted using a Bruker D2Phaser diffractometer to investigate the mineralogical composition and crystalline structures formed as a result of geopolymerization processes. Prior to analysis, the samples were finely ground to a uniform powder using a IKA A11 analytical mill to ensure homogeneity and minimize preferred orientation effects.

The powder samples were placed in sample holder with minimal compaction to provide a smooth surface. Measurements were performed using Cu Kα radiation (wavelength *λ* = 1.5406 Å) over a 2*θ* range of 10° to 80°, with a step size of 0.02° and a dwell time of 0.1 seconds per step. The resulting diffractograms were processed using HighScore Plus software, and crystalline phases were identified through comparison with the ICDD PDF-2 database.

### 2.9. Optical microscopy and SEM analysis for pore structure visualization

The porous structure of the obtained foamed materials was investigated using the method of optical microscopy. The studies were carried out at the “Nanomaterials and Nanotechnologies” Research Laboratory of ASOIU using an AmScope ME520 Trinocular Microscope. The porous structure was examined at magnifications of 10×, 20×, 40×, and 100×.

The microstructural analysis of the foamed geopolymer samples was carried out using a TESCAN VEGA 3 scanning electron microscope. Prior to imaging, the samples were fractured to expose fresh cross-sections and then dried at 60 °C for 24 hours to eliminate residual moisture. Conductive coating was not applied, as the inherent composition of the geopolymer matrix provided sufficient surface conductivity for SEM observation. SEM analysis was performed in high-vacuum mode using a secondary electron detector (BSE) with accelerating voltage 5 kV, in order to prevent overcharging inside the chamber.

### 2.10. Thermal analysis (TGA-DTA)

Thermal behaviour of the synthesized geopolymer was investigated using a simultaneous thermal analyser PerkinElmer STA 6000, at the Institute of Geology and Geophysics of the Azerbaijan National Academy of Sciences (ANAS). The analysis was carried out in the temperature range of 20–1000 °C under a nitrogen atmosphere with a constant flow rate of 50 mL min^−1^ in order to prevent oxidation. Approximately 10 mg of finely powdered sample was placed in an open alumina crucible, and the heating rate was maintained at 10°C min^−1^ throughout the test.

## 3. Results and discussion

### 3.1. Chemistry and probable mechanism of geopolymerization in salt-activated systems

The geopolymerization process in traditional systems is typically driven by highly alkaline or strongly acidic solutions, which facilitate the dissolution of reactive aluminosilicate phases and their subsequent reorganization into three-dimensional gel networks. In this work, a fundamentally different activation route was employed: salt activation using sodium dihydrogen phosphate (NaH_2_PO_4_). This method offers hybrid activation pathway that simultaneously involves both aluminosilicate and phosphate components, yielding a novel amorphous geopolymeric matrix.

The WB precursor is primarily consisting of:

– SiO_2_ (52.67 wt%);

– Al_2_O_3_ (15.6 wt%);

– CaO (8.47 wt%).

Only a fraction of these phases is chemically reactive. Mullite and quartz are relatively inert, while feldspars and amorphous aluminosilicate glass are prone to partial dissolution under activation.

When aqueous solution of Ac (NaH_2_PO_4_) is introduced into WB, the following equilibria occur:

Dissociation of salt:

8 NaH_2_PO_4_ → Na^+^ + H_2_PO_4_^−^

9 H_2_PO_4_^−^ ↔ HPO_4_^2−^ + H^+^

This solution (pH = 5.5–7) facilitates the partial breakdown of reactive aluminosilicates *via* proton-assisted hydrolysis:

10 Al_2_O_3_ SiO_2_ + H^+^ + 5H_2_O → 2Al(OH)_3_ + Si(OH)_4_ + H^+^

11 CaAl_2_Si_2_O_8_ + 2H^+^ + 6H_2_O → 2Al(OH)_3_ + 2Si(OH)_4_ + Ca^2+^

The dissolution releases aluminium and silicon hydroxide species into the solution which are essential for subsequent polymerization steps.

Polycondensation and gel network formation:

Once in solution, reactive species undergo a series of condensation reactions leading to the formation of an amorphous polymeric network:

12 *n*Al(OH)_3_ + *n*H_2_PO_4_^−^ + *n*H^+^ + *n*H_2_O → [(HO)_3_–Al–O–P–(OH)_3_]_*n*_^−^ + *n*H_2_O

13 *n*Si(OH)_4_ + H_2_PO_4_^−^ + 2*n*H^+^ → [(HO)_3_–Si–O–P–(OH)_3_]_*n*_^−^ + *n*H_2_O

These reactions produce Al–O–P and Si–O–P bridges, characteristic of phosphate-containing geopolymeric gels.

14 *n*Al(OH)_4_^−^ + *n*Si(OH)_4_ → [(HO)_3_–Al–O–Si–(OH)_3_]_*n*_ + *n*H_2_O

15 *n*Si(OH)_4_ + *n*Si(OH)_4_ → [(HO)_3_–Si–O–Si−(OH)_3_]_*n*_ + *n*H_2_O

These reactions are characteristic of silicoaluminate framework condensation (traditional geopolymer bonds).

Sodium ions act as charge-balancing species for AlO_4_ tetrahedra within the network, stabilizing the gel structure:

16 [AlO_4_]^−^ + Na^+^ → [AlO_4_Na]

The resulting amorphous matrix is characterized by a three-dimensional network of interconnected Si–O–Al, P–O–Al, and P–O–Si bridges, stabilized by Na^+^ cations. This hybrid gel is fundamentally different from both conventional alkali-activated (N-A-S-H type)^40^ and phosphate-activated (AlPO_4_ type)^41^ geopolymers.

Proposed mechanism of a novel-type of geopolymerization was confirmed by FTIR and XRD analyses and discussed in parts 3.7 and 3.8.

### 3.2. Effect of foaming agent and activator concentration on bulk density

The bulk density of foamed geopolymers is a critical characteristic that determines the effectiveness of the materials as insulating medium. It is directly influenced by the degree of porosity gained during the foaming process. In the present work, bulk density was used as a primary indicator to assess the efficiency of foaming as a function of citric acid concentration and the mass content of limestone.

**Fig. 4:**
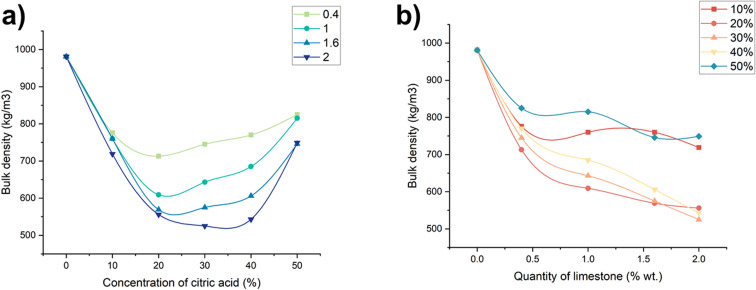
Dependence of bulk density change on (a) foaming activator concentration; (b) foaming agent content (concentration of salt activator solution 60%, WB to Ac ratio is 50 : 50 wt%).

It was observed that the introduction of even a small amount of limestone (0.4 wt%) and a citric acid solution (10 wt%) led to a significant reduction in bulk density from 1656 kg m^−3^ (monolithic geopolymer) to 776 kg m^−3^ (Fig. 4). This sharp decline is attributed to the acid–carbonate reaction between citric acid and CaCO_3_, which generates CO_2_ gas that becomes retained in the matrix during hardening, thus forming porosity.

Further increasing the concentration of citric acid to 20 wt% reduced the bulk density even more significantly, depending on the limestone content. For example, at 2 wt% limestone and 20 wt% citric acid, the bulk density decreased to approximately 569 kg m^−3^. However, a continued increase in citric acid concentration beyond this point (*e.g.*, 30–50 wt%) began to reverse the trend: bulk density increased, reaching values between 746–815 kg m^−3^.

This reverse tendency can be explained by the mismatch between the rate of gas evolution and the viscosity development of the geopolymer paste.

At higher acid concentrations, gas evolution occurs more rapidly, but the viscosity of the system may still be too low to effectively retain the CO_2_ bubbles. As a result, a portion of the gas degassed before solidification, leading to a lower gas bubbles retention efficiency and hence a higher bulk density.

Furthermore, the optimal foaming effect – characterized by low bulk density and stable pore structure – was found to occur at a citric acid concentration of 30 wt% and limestone content of 2 wt%. Under these conditions, the gas evolution and the structural setting rate are well-balanced, maximizing pore formation and gas bubbles retention.

### 3.3. True, open and closed porosities

Porosity plays a fundamental role in determining the functional properties of porous materials, especially for applications involving thermal insulation, fluid permeability, and mechanical resistance. In this study, three types of porosity were evaluated: true (total) porosity, open porosity (connected to the surface and enabling fluid flow), and closed porosity (isolated voids).

**Fig. 5:**
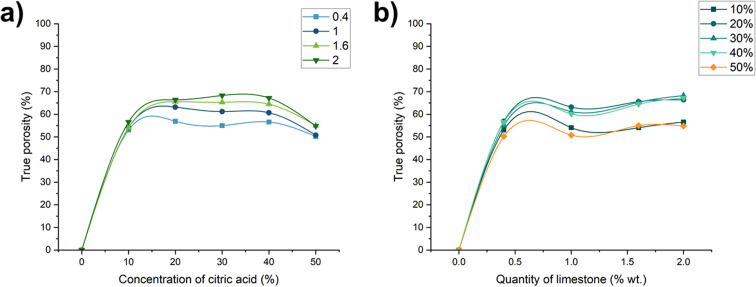
Dependence of true porosity change on (a) foaming activator concentration; (b) foaming agent content (concentration of salt activator solution 60%, WB to Ac ratio is 50 : 50 wt%).

True porosity (Fig. 5) increased with both the concentration of citric acid and the content of limestone, up to optimal point. At 30 wt% citric acid and 2 wt% limestone, the maximum true porosity was observed at 68.3%. This is significant enhancement compared to the monolithic geopolymer sample, confirming the high efficiency of foaming achieved under salt activation.

**Fig. 6:**
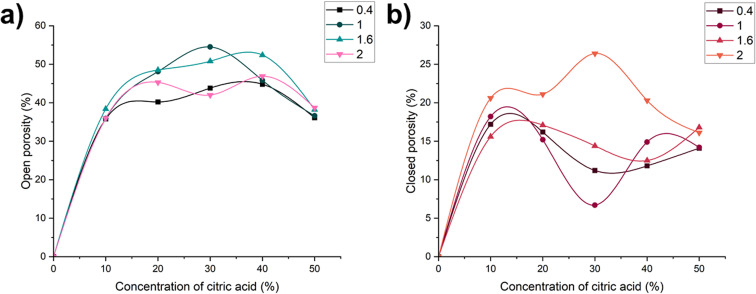
Dependence of (a) open porosity and (b) closed porosity change on foaming activator concentration (concentration of salt activator solution 60 WB to Ac ratio is 50 : 50 wt%).

The variation of open and closed porosity was also analysed (Fig. 6). It was found that open porosity dominated in samples with lower limestone content (0.4–1.6 wt%) and moderate citric acid concentrations (20–30 wt%), reaching values of ∼50%. Closed porosity, on the other hand, was maximized in samples with 2 wt% limestone and 30% citric acid concentration. This composition facilitated the formation of enclosed pores, essential for reducing thermal conductivity.

Interestingly, both very low (10 wt%) and very high (50 wt%) concentrations of citric acid yielded lower closed porosity. At low concentrations, insufficient gas generation limits pore formation. At excessively high concentrations, the gas production rate exceeds the ability of the matrix to retain gas, resulting in premature degassing of CO_2_ and insufficient forming of the porous structure.

These findings show that balancing foaming kinetics and matrix setting behaviour is crucial to achieve a desirable pore structure with high insulation potential.

### 3.4. Mechanical properties and structural integrity

Mechanical strength, particularly compressive strength, is a critical criterion for the practical application of porous geopolymer materials. In this study, compressive strength tests were performed on foamed samples with varying limestone and citric acid contents. The results demonstrate a clear inverse relationship between porosity and compressive strength.

**Fig. 7:**
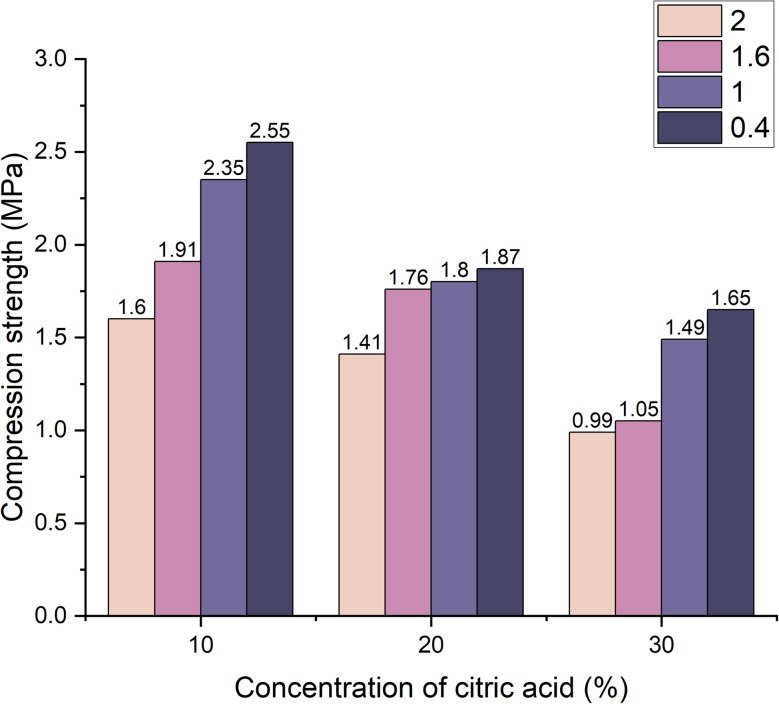
Dependence of compressive strength change on foaming activator concentration during 7 days of curing (concentration of salt activator solution 60%, WB to Ac ratio is 50 : 50 wt%).

The monolithic reference sample displayed the highest compressive strength at 9.37 MPa. In foamed samples tested after 7 days (Fig. 7), strength values ranged from 0.4 MPa to 2.1 MPa.

The highest strength was observed in samples containing 0.4 wt% limestone and 20 wt% citric acid. As limestone content increased to 2 wt% and citric acid concentration exceeded 30 wt% compressive strength significantly decreased due to the formation of larger, more irregular pores and failure of material structure integrity.

**Fig. 8:**
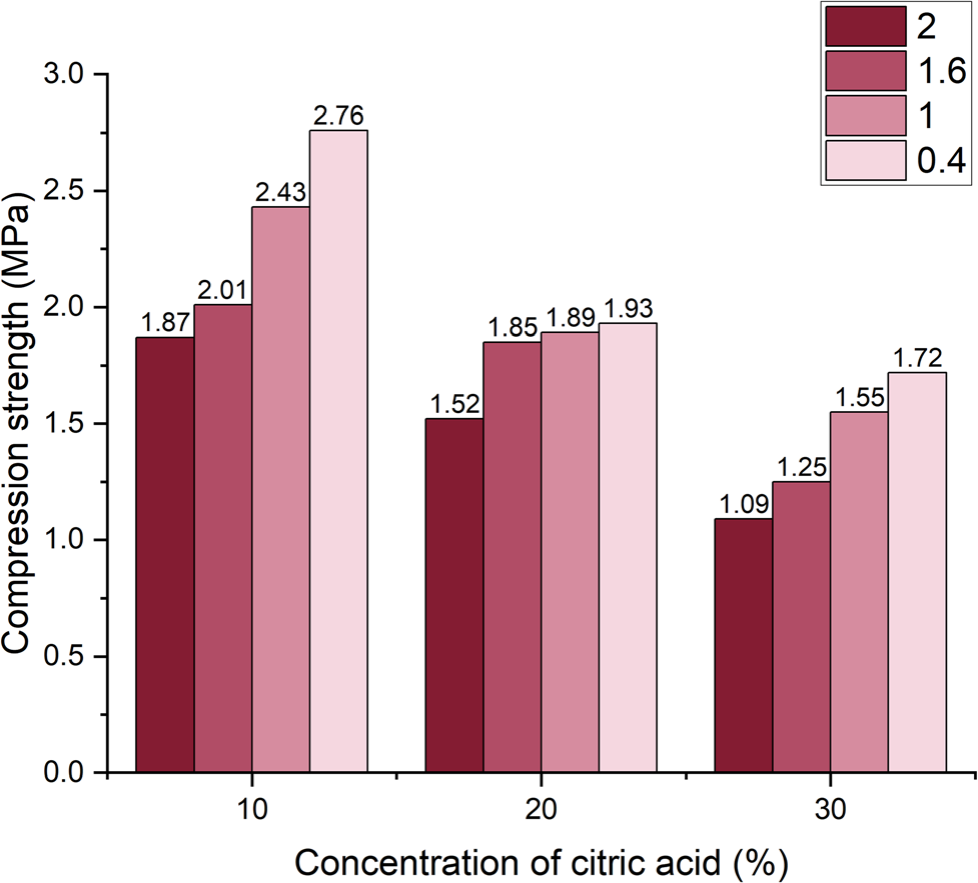
Dependence of compressive strength change on foaming activator concentration during 14 days of curing (concentration of salt activator solution 60%, WB to Ac ratio is 50 : 50 wt%).

To assess strength evolution over time, additional compressive strength tests were conducted at 14 and 28 days. At 14 days (Fig. 8), the trend observed at 7 days persisted: the lowest strength (1.09 MPa) was recorded for the sample with 30 wt% citric acid and 2 wt% limestone, while the highest strength (2.76 MPa) was measured in the sample with 10 wt% citric acid and 0.4 wt% limestone.

**Fig. 9:**
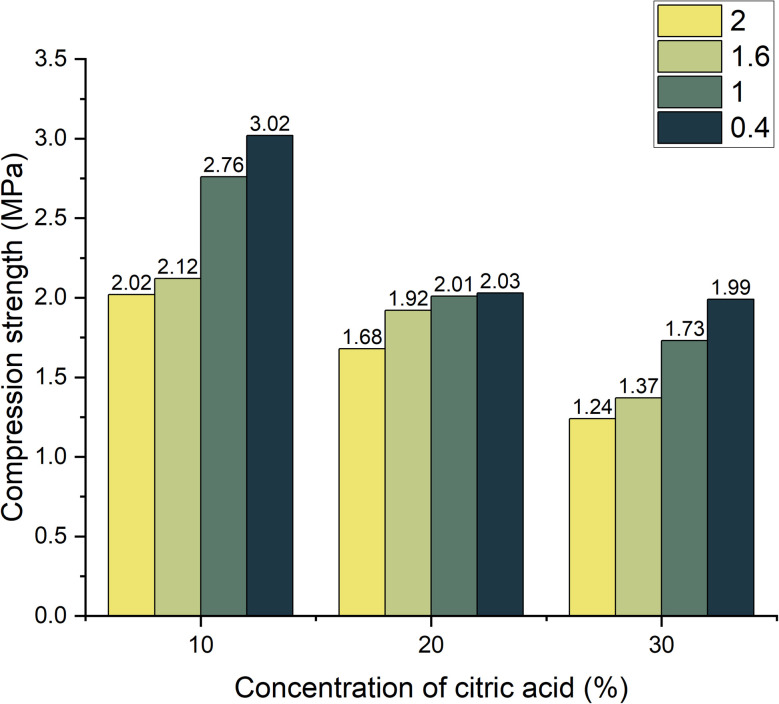
Dependence of compressive strength change on foaming activator concentration during 28 days of curing (concentration of salt activator solution 60%, WB to Ac ratio is 50 : 50 wt%).

Similarly, at 28 days (Fig. 9), the lowest strength remained in the sample with high-foaming level (1.24 MPa) and the highest in the composite with low-foaming level (3.02 MPa). This progressive increase in strength with curing time across all compositions suggests ongoing geopolymerization and consolidation of structure.

**Fig. 10:**
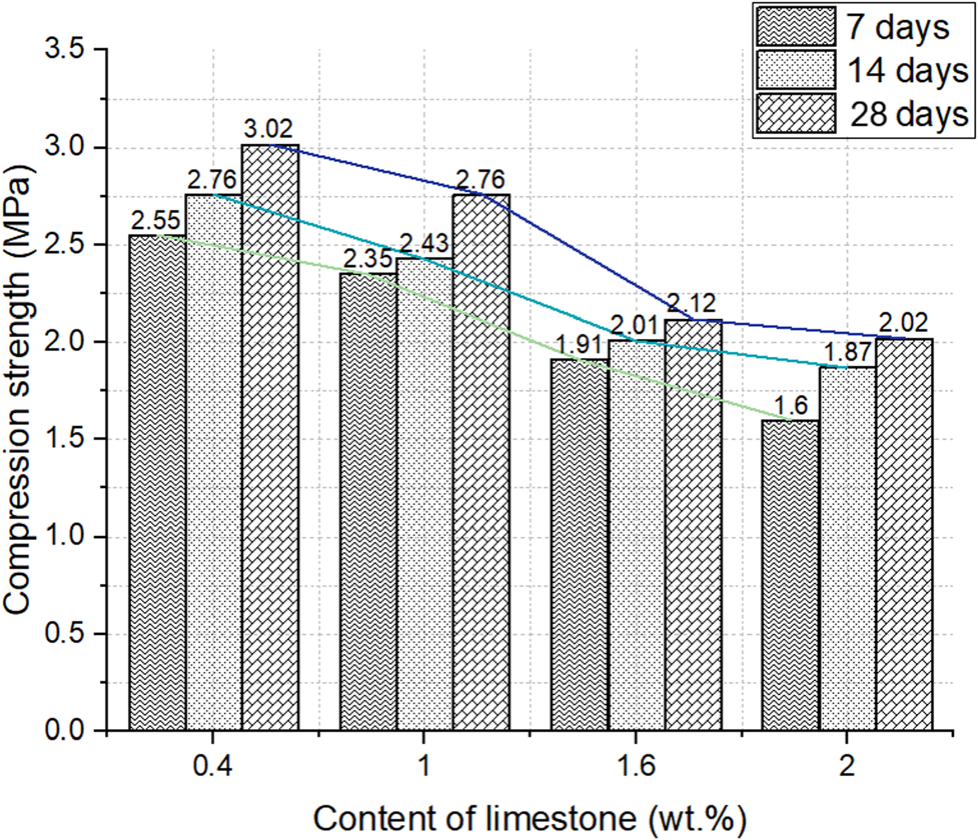
Dependence of compressive strength change on content of foaming agent during 28 days of curing (concentration of salt activator solution 60%, WB to Ac ratio is 50 : 50 wt%).

However, the relative differences between compositions remain consistent. The data confirm that highly foamed structures with large pores continue to exhibit lower strength even after extended curing, whereas moderately foamed samples retain superior mechanical integrity over time (Fig. 10).

These results underline the need to enhance foaming parameters to strike a balance between low bulk density (high porosity) and adequate mechanical properties. In application where load-bearing is secondary to insulation, materials with moderate porosity (∼60–65%) and strength ∼1.5–2.5 MPa after 28 days may provide the best trade-off.

### 3.5. Research of thermal insulation of porous geopolymer materials

**Fig. 11:**
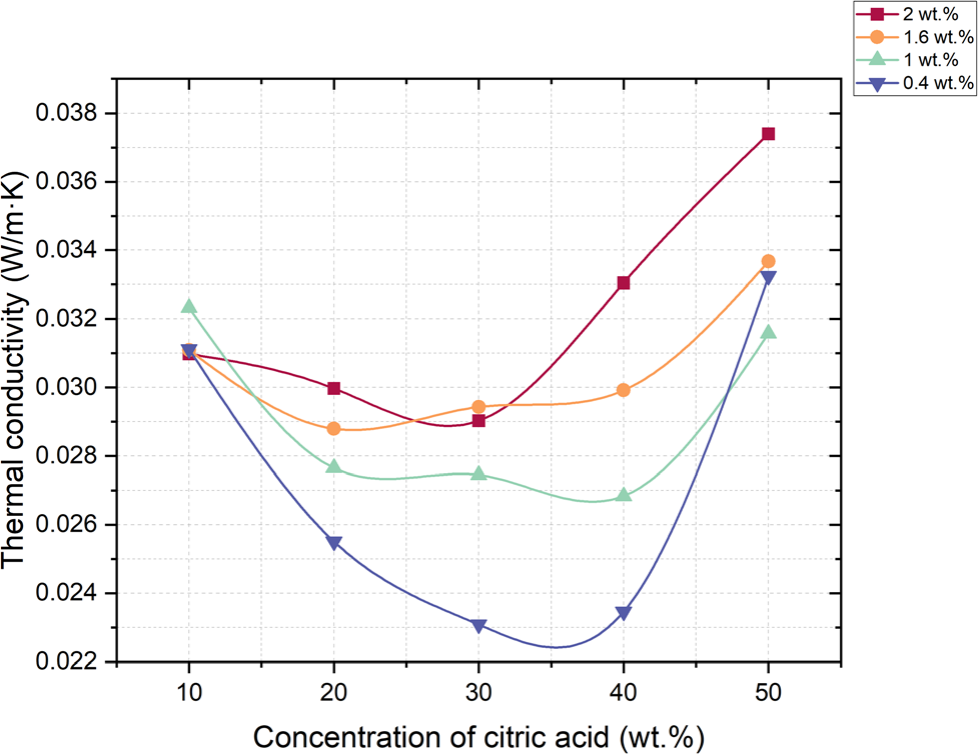
Dependence of thermal conductivity coefficient change on foaming activator concentration at 50 °C (concentration of salt activator solution 60%, WB to Ac ratio is 50 : 50 wt%).

**Fig. 12:**
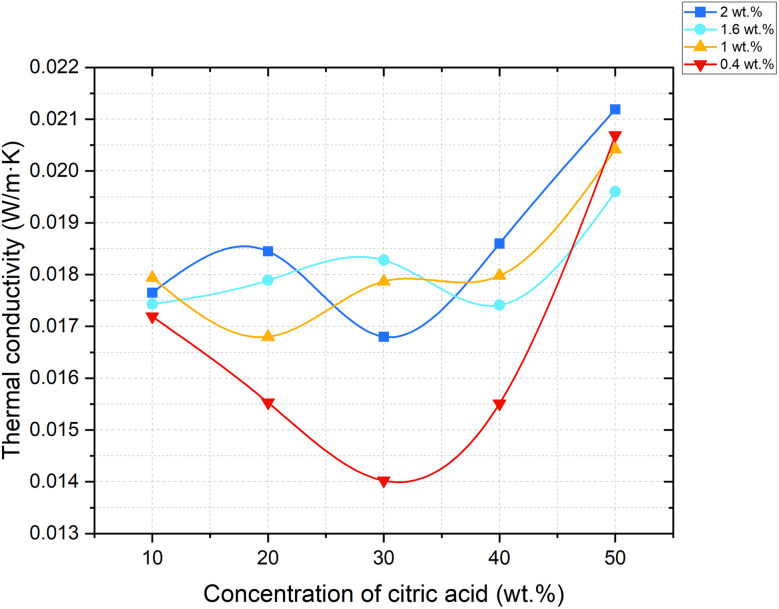
Dependence of thermal conductivity coefficient change on foaming activator concentration at 70 °C (concentration of salt activator solution 60%, WB to Ac ratio is 50 : 50 wt%).

**Fig. 13:**
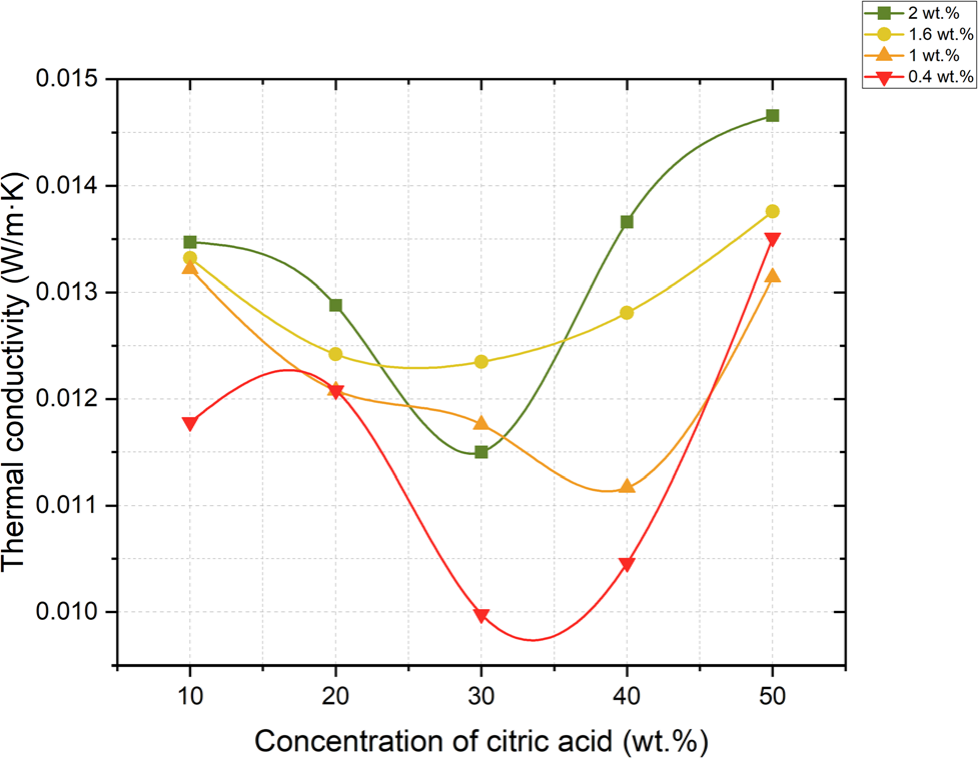
Dependence of thermal conductivity coefficient change on foaming activator concentration at 90 °C (concentration of salt activator solution 60%, WB to Ac ratio is 50 : 50 wt%).

In this study, thermal conductivity was measured using a steady-state longitudinal heat flow method at three controlled temperatures: 50 °C (Fig. 11), 70 °C (Fig. 12), and 90 °C (Fig. 13). Samples were pre-dried to remove residual moisture, and test specimens were selected based on a range of limestone and citric acid contents to assess how porosity and morphology influence insulation properties.

He results showed a consistent trend across all temperature points: samples with lower bulk density and higher closed porosity exhibited significantly reduced thermal conductivity. The lowest value was observed in the sample containing 0.4 wt% limestone and 30 wt% citric acid, which achieved a thermal conductivity of just 0.00998 W (m K)^−1^ at 90 °C. This value positions the material among the most effective thermal insulators, comparable to or even outperforming materials like expanded polystyrene (EPS), polyurethane foams, and mineral wool. This excellent performance can be attributed to a high degree of closed-cell porosity in the matrix. Closed pores containing CO_2_ – a gas with low thermal conductivity – reduce the conduction and transferring of heat through the material.

The small size and even distribution of these pores prevent the formation of continuous heat transfer paths, thereby minimizing overall thermal conductivity. Conversely, samples with more open or interconnected pores, especially those foamed with higher citric acid concentrations (40–50 wt%), displayed higher thermal conductivity values. This is due to the increased air movement and convective heat transfer within the material.

The thermal conductivity results presented in Table 4 demonstrate a clear dependence on both the composition of the foamed geopolymer and the measurement temperature.

**Table 4:** Thermal conductivity (W (m K)^−1^) of foamed geopolymer samples at various temperatures

Temperature, °C	Conc. of citric acid, wt%	Quantities of limestone, wt%			
		2	1.6	1	0.4
50	10	0.03098	0.03109	0.03233	0.03112
	20	0.02997	0.02879	0.02765	0.02551
	30	0.02903	0.02943	0.02745	0.02308
	40	0.03305	0.02992	0.02684	0.02346
	50	0.0374	0.03367	0.03158	0.03324
70	10	0.01765	0.01743	0.01794	0.01719
	20	0.01845	0.01789	0.0168	0.01553
	30	0.0168	0.01828	0.01787	0.01402
	40	0.0186	0.01741	0.01798	0.01551
	50	0.02119	0.0196	0.02042	0.02069
90	10	0.01347	0.01332	0.01322	0.01178
	20	0.01288	0.01242	0.01208	0.01208
	30	0.0115	0.01235	0.01176	0.00998
	40	0.01366	0.01281	0.01117	0.01046
	50	0.01466	0.01376	0.01314	0.01351

These findings clearly demonstrate that the synthesis conditions – particularly the foaming activator concentration and the amount of foaming agent – must be finely tuned to generate the optimal closed-cell porous structure. Geopolymers produced with salt activation under controlled foaming exhibit not only low conductivity but also ecological advantages due to their waste-based origin and low-carbon synthesis route.

### 3.6. Microstructural observations and morphology

**Fig. 14:**
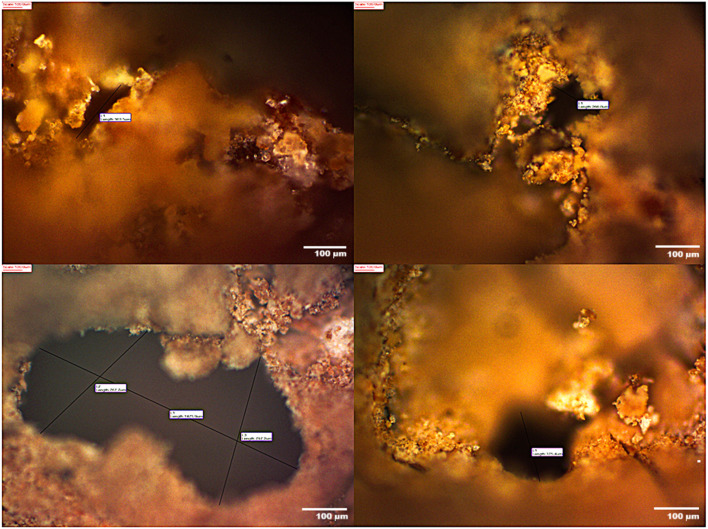
Microstructural morphology of salt-activated foamed geopolymers observed *via* optical microscopy.

Optical microscopy was employed to examine the microstructure of the foamed geopolymer samples at various magnifications (Fig. 14). The best-performing samples in terms of thermal and mechanical properties displayed spherical, homogeneously distributed pores ranging from 256 to 1475 μm in diameter. These structures were indicative of stable gas retention and uniform expansion during the foaming process.

**Fig. 15:**
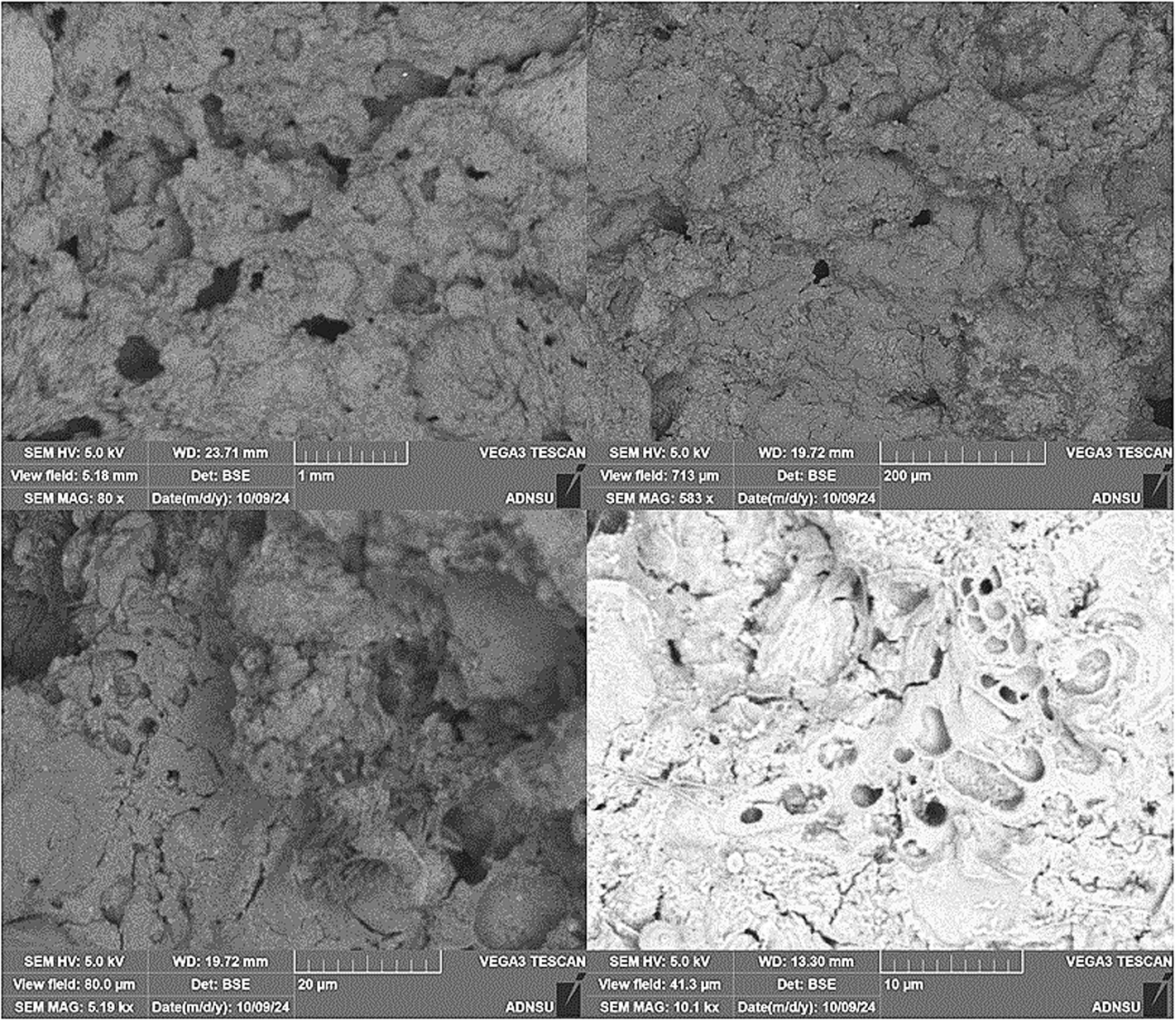
Microstructural morphology of salt-activated foamed geopolymers observed *via* Scanning Electron Microscopy (SEM).

The morphology of the foamed geopolymer samples was investigated by scanning electron microscopy (SEM) (Fig. 15). The SEM image clearly shows rounded pores ranging from approximately 10 to 60 μm in diameter, forming a loose, disordered structure. Such a coarse pore structure is attributed to the use of a very high concentrations of citric acid (50%) in combination with 2 wt% CaCO_3_. The acid-carbonate reaction leads to the rapid evolution of CO_2_, resulting in the formation of large gas bubbles.

The image with SEM magnification 5.19k×, scale bar 20 μm, reveals a much more uniform and compact microstructure. The pores are smaller (ranging from 5 to 20 μm) and evenly distributed throughout the matrix. This figure is related to the sample with a low amount of foaming agent (1 wt%) and low concentrations of foaming activator (30 wt%). In this case, less CO_2_ is released, and the resulting bubbles are smaller and more stable. The reaction occurs in a more controlled mode, which allows the developing geopolymer paste to retain the gas bubbles more effectively without excessive coalescence.

The image with SEM magnification 10.1k×, scale bar 10 μm exhibits a microporous morphology with pores ranging from 0.3 to 2 μm. No large open pores are visible, and the matrix appears densely packed. This figure is related to the sample with a low amount of foaming agent (0.4 wt%) and low concentrations of foaming activator (10 wt%). In this case, only a limited amount of gas is generated, resulting in a geopolymer structure dominated by closed gel pores and nanovoids. The absence of significant gas evolution during curing leads to a solidified matrix with minimal porosity and high homogeneity.

Overall, SEM analysis and optical microscopy demonstrated that the microstructure of foamed geopolymers can be effectively tuned by adjusting the concentrations of citric acid and CaCO_3_. Highly porous samples are ideal for insulation applications, offering low weight and good thermal properties but limited structural capacity. Dense samples, conversely, are more suitable for high load-bearing.

### 3.7. FTIR spectral analysis of salt-activated geopolymer

**Fig. 16:**
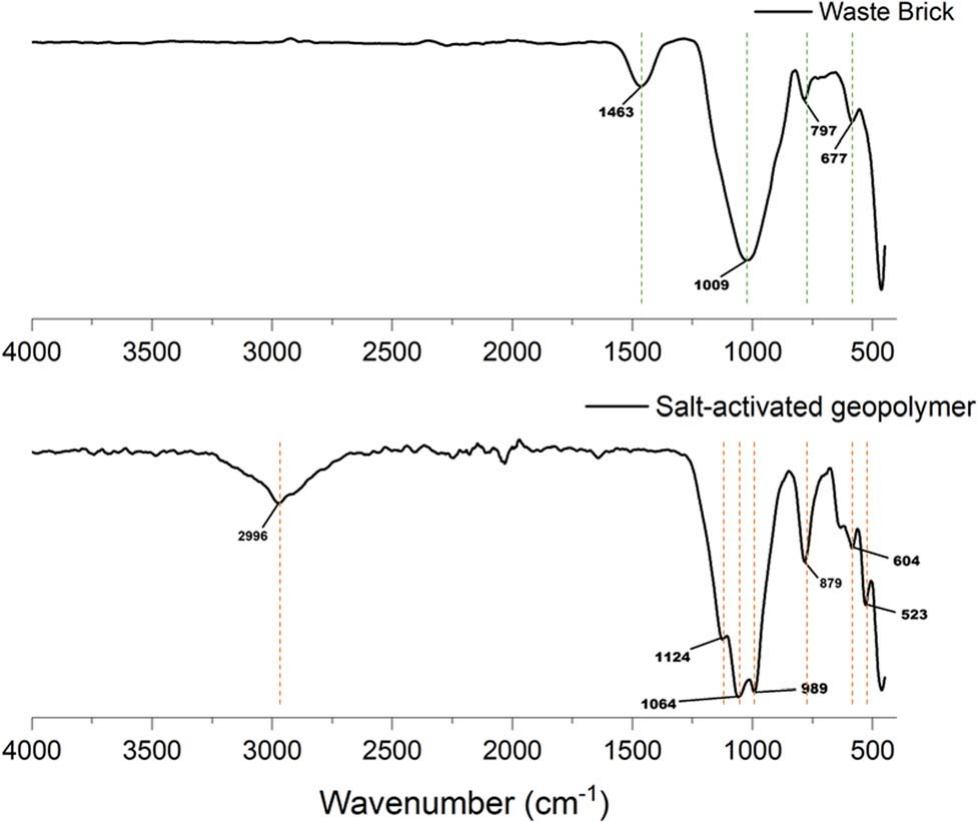
Comparative FTIR spetra of waste brick and salt-activated geopolymer.

The FTIR spectra of the WB precursor and salt-activated geopolymer (Fig. 16) clearly illustrates the chemical transformation caused by activation with Ac (NaH_2_PO_4_). The spectrum of WB is dominated by bands typical of fired ceramic materials containing quartz, feldsars, and mullite. A peak at 1009 cm^−1^ corresponds to the asymmetric stretching of Si–O–Si in crystalline quartz and feldspar phases,^14,36^ while the peaks at 797 and 677 cm^−1^ reflect symmetric Si–O–Si stretching and O–Si–O bending modes, characteristic of crystalline quartz.^37^ A weaker band at 1463 cm^−1^ can be attributed to carbonate impurities, likely derived from residual calcite or limestone. Together, these features indicate that WB is largely crystalline, with limited amorphous aluminosilicate content available for reaction.

After activation, the FTIR spectrum of the salt-activated geopolymer undergoes substantial changes, confirming the breakdown of reactive WB phases and the formation of a new amorphous network. The original quartz band at 1009 cm^−1^ largely disappears and is replaced by a broad, intense peak centered at 1124 cm^−1^ (Si–O–Si) with shoulders near 1064 cm^−1^ (Si–O–Al) and 989 cm^−1^ (P–O–Si). This shift to higher wavenumbers indicates the partial dissolution of feldspar and mullite under conditions of NaH_2_PO_4_ and geopolymerization of dissolved species into a gel where aluminum substitutes into the silicate framework. The position and breadth of this band also reflect the superposition of Si–O–Al vibrations for the newly formed sodium aluminosolicate hydrate (N-A-S-H) gel with P–O–Si vibrations created by phosphate integration.^38^

A new peak emerges approximately at 879 cm^−1^, which is asigned to Al–O–P stretching. This band provides strong evidence that phosphate groups from the NaH_2_PO_4_ activator have chemically bonded to tetrahedral environments, forming phosphate-alumina linkages.^38^ The transformation is further supported by the appearance of a peak at 604 cm^−1^, associated with Na–O–Al bending vibrations in NaAlO_4_ tetrahedra.^35^ These sodium-aluminate units demonstrate that sodium ions from the activator have been incorporated into the gel to charge-balance AlO_4_^−^ tetrahedra, stabilizing the geopolymeric network.^14^ Another band at 523 cm^−1^ reflects Si–O–Al bending and octahedral Al deformation, confirming the condensation of aluminosilicate species into a denser three-dimensional gel structure.^14,36^

Several additional changes strengthen the interpretation of structural transformation. The carbonate band at 1463 cm^−1^, visible in the WB spectrum, disappears completely, suggesting that residual calcite reacted fully during the geopolymerization process. Meanwhile, a weak signal at approximately 2996 cm^−1^ appears in the geopolymer spectrum, likely due to minor C–H stretching from residual organics or traces of adsorbed CO_2_ during curing.

The comparative analysis of these spectra reveals the mechanism of transformation: feldspar and mullite components in WB undergone partial dissolution, releasing Al(OH)_4_^−^ and Si(OH)_4_ species into solution. These reactive species then condense with the phosphate ions (H_2_PO_4_^−^) from the activator, creating a hybrid gel network that integrates Si–O–Al bonds typical of aluminosilicate geopolymers, P–O–Si and Al–O–P bridges derived from phosphate polymerization, and Na–O–Al linkages from sodium-aluminate species. Quartz bands at 797 and 677 cm^−1^ remain diminished but detectable, indicating that quartz survives activation largely as an inert filler. Overall, the FTIR evidece confirms that NaH_2_PO_4_ converts the mostly crystalline WB into an amorphous hybrid phosphate-aluminosilicate structure, where Si–O–Al, P–O–Si, Al–O–P, and Na–O–Al units coexist. This hybridization demonstrates a dual geopolymerization mechanism, combining classical aluminosilicate gelation with phosphate cross-linking, and highlights the structural uniqueness of the salt-activated geopolymer compared to conventional alkali- or phosphate-activated systems.

### 3.8. XRD analysis and phase composition

**Fig. 17:**
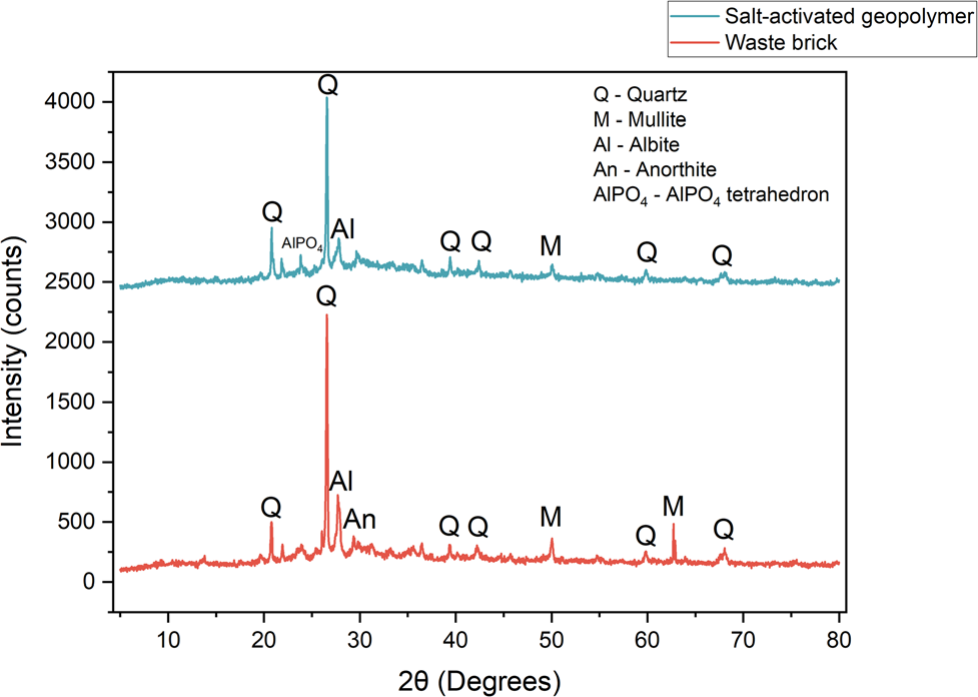
Comparative XRD analysis of waste brick and salt-activated geopolymer.

Fig. 17 represent the XRD pattern of waste brick. The waste brick shows strong, sharp XRD peaks that match crystalline quartz (SiO_2_) and alkali/calcium feldspar minerals (albite NaAlSi_3_O_8_, anorthite CaAl_2_Si_2_O_8_). These phases dominate the pattern, indicating a mature fired clay structure. Secondary peaks are attributed to iron oxides (hematite Fe_2_O_3_) and minor silicates such as diopside (MgCaSi_2_O_6_) and mullite (3Al_2_O_3_·2SiO_2_). Although the brick is largely crystalline, the firing process converts most clay minerals into a glassy amorphous aluminosilicate matrix. This amorphous glass phase may appear as a weak broad “hump” or raised background (typically 15–30° 2*θ*) underlying the sharp peaks. The sharp, high-intensity peaks (narrow full-widths) indicate the waste brick has a high degree of crystallinity for the identified phases. The distinct reflections (quartz (101) at 26.6° 2*θ*) show well-ordered lattices.

After salt activation, the XRD pattern still contains peaks assignable to the original silicate minerals, but their intensities and sharpness are altered. In particular, quartz peaks remain visible, suggesting that much of the α-quartz is unchanged by the activation step. In contrast, peaks due to reactive feldspars or mullite are significantly reduced or absent, implying partial dissolution of these aluminosilicate precursors during activation. New crystalline reflections may appear, for example, AlPO_4_ is present with peaks near 20.9–55.1° 2*θ*. Such new peaks would indicate the formation of new structures during geopolymerization.

Comparing the patterns shows that quartz (SiO_2_) is largely preserved after activation, whereas many feldspars and mullite peaks have diminished or vanished. This indicates that refractory quartz grains acted as inert filler, whereas more soluble aluminosilicates (anorthite, albite, and mullite) were at least partially dissolved by salt solution, and simultaneously formed new tetrahedrons (AlPO_4_).

XRD analysis confirmed the formation of a fundamentally new structural composition in the salt-activated geopolymer, distinct from both conventional alkali-activated and phosphate-activated geopolymers. Compared to the raw material (waste brick), the activated geopolymer exhibited a significant reduction in crystalline feldspar and mullite phases, along with the appearance of a broad amorphous hump typical of N-A-S-H type gels. The partial preservation of quartz, the dissolution of reactive aluminosilicates, and the emergence of additional phases such as AlPO_4_ indicate a complex, hybrid structure formed through simultaneous alkaline and phosphate-type interactions. These suggests that the salt-based activation route promotes a unique geopolymerization mechanism, yielding a mixed aluminosilicate–phosphate amorphous matrix with the properties and structure not observed in traditional systems.

### 3.9. Thermal behaviour analysis (TGA/DTA)

**Fig. 18:**
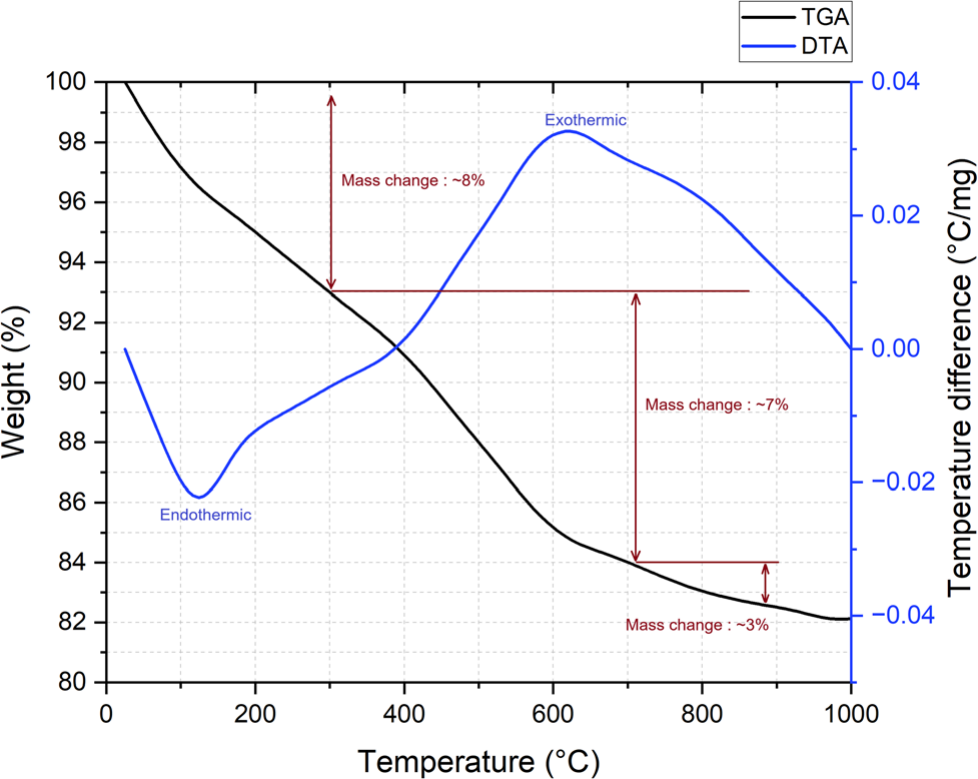
TGA/DTA curve of the salt-activated geopolymer.

The thermal behaviour of the geopolymer sample activated with sodium dihydrogen phosphate was investigated using thermogravimetric analysis (TGA) and differential thermal analysis (DTA), as shown in Fig. 18. The TGA curve indicates a three-step weight loss up to 1000 °C, while the DTA curve reveals both endothermic and exothermic events associate with these transformations.

The first stage of mass loss, occurring between room temperature and ∼300 °C, is attributed to the evaporation of physically adsorbed and loosely bound water within geopolymer matrix. This stage corresponds to a mass loss of approximately 8%, and is accompanied by a broad endothermic peak on the DTA curve centred around 120 °C. This behaviour is consistent with the dehydration events observed in phosphate-based geopolymers and other hydrous ceramic systems.^38^

The second stage, ranging from 300 to 700 °C, shows an additional 7% mass loss, likely due to the removal of chemically bonded hydroxyl groups and further condensation reactions within the geopolymer structure. During this phase the DTA curve displays a pronounced exothermic peak near 700 °C, which may be associated with the crystallization of aluminosilicate or related phases, a common phenomenon in alkali geopolymer systems.^39^

The third stage, between 700 and 1000 °C, accounts for a further ∼3% weight loss. This may result from the transformation of amorphous phases into more stable crystalline structures, accompanied by minor gas release or structural rearrangements. The total weight loss observed for the salt-activated geopolymer sample (∼18%) places it between the typical ranges of alkali- and phosphate-based systems. The presence of a defined exothermic peak and moderate weight retention above 700 °C indicates the formation of thermally stable crystalline phases, suggesting potential for high-temperature applications such as fire-resistant materials or refractory binders.

## 4. Conclusion

(1) It was experimentally confirmed that salt activation using sodium dihydrogen phosphate (NaH_2_PO_4_) enables effective geopolymerization of ceramic waste (waste brick) and allows for the formation of porous, foamed structures suitable for thermal insulation applications;

(2) It was established that the apparent (bulk) density of geopolymer foams can be purposefully regulated by adjusting the concentration of the foaming activator (citric acid) and the quantity of the foaming agent (limestone). The minimal bulk density obtained was 525 kg m^−3^ at a citric acid concentration of 30% and limestone content of 2 %wt., which is approximately 3.2 times lower than the monolithic sample (1656 kg m^−3^).

(3) It was determined that the maximum true porosity of foamed samples reached 68.3% at 30% citric acid and 2% wt limestone. This value is over four times higher than the porosity of the monolith. The porosity values were found to be inversely proportional to the bulk density of the samples.

(4) It was determined that the compressive strength of foamed geopolymer samples varies between 0.4 to 3.02 MPa, depending on the amount of foaming agent, citric acid concentration, and time of curing. The maximum compressive strength (9.37 MPa) was recorded for monolithic samples.

(5) It was established that thermal conductivity measurements confirmed the insulating character of the materials: samples with the highest closed porosity demonstrated the lowest thermal conductivity (0.00998 W (m K)^−1^), which correlates with the presence of gas-filled, isolated pores.

(6) It was proven that by changing the concentration of foaming activator and the amount of limestone, it is possible to tailor the bulk density, porosity type, mechanical strength and thermal insulation properties of geopolymer foams, making them suitable for practical applications as thermal insulation materials.

## Conflicts of interest

There are no conflicts to declare.

## Supplementary Material

RA-015-D5RA05707H-s001

## Data Availability

The authors confirm that the data supporting the findings of this study are available within the article and SI. All data supporting the findings of this study, including experimental, microscopic images (optical and SEM), and analytical data, are provided within the main article. Additional data (XRD patterns) are included in the SI. Supplementary information is available. See DOI: https://doi.org/10.1039/d5ra05707h.
