# Supplementary material for: Synthesis and foaming of a novel type of porous geopolymer material via salt activation
Source: RSC Adv. 2025 Oct 21;15(47):39832–46. doi: 10.1039/d5ra05707h (PMC12538389; doi:10.1039/d5ra05707h)
Supplement: RA-015-D5RA05707H-s001 [file RA-015-D5RA05707H-s001.pdf]

Supplementary Information (SI) for RSC Advances.
This journal is © The Royal Society of Chemistry 2025

Synthesis and foaming of a novel type of porous geopolymer materials via salt activation.

Goryunova Kristina [a], Gahramanli Yunis [a]
Department of "Chemistry and Inorganic Substances Technology", Azerbaijan State Oil and Industry University, Baku city.

E-mail: kristina.qoryunova.i@asiou.edu.az

Table of Contents:

1. XRD pattern of Waste Brick;
2. XRD pattern of Salt-Activated Geopolymer.

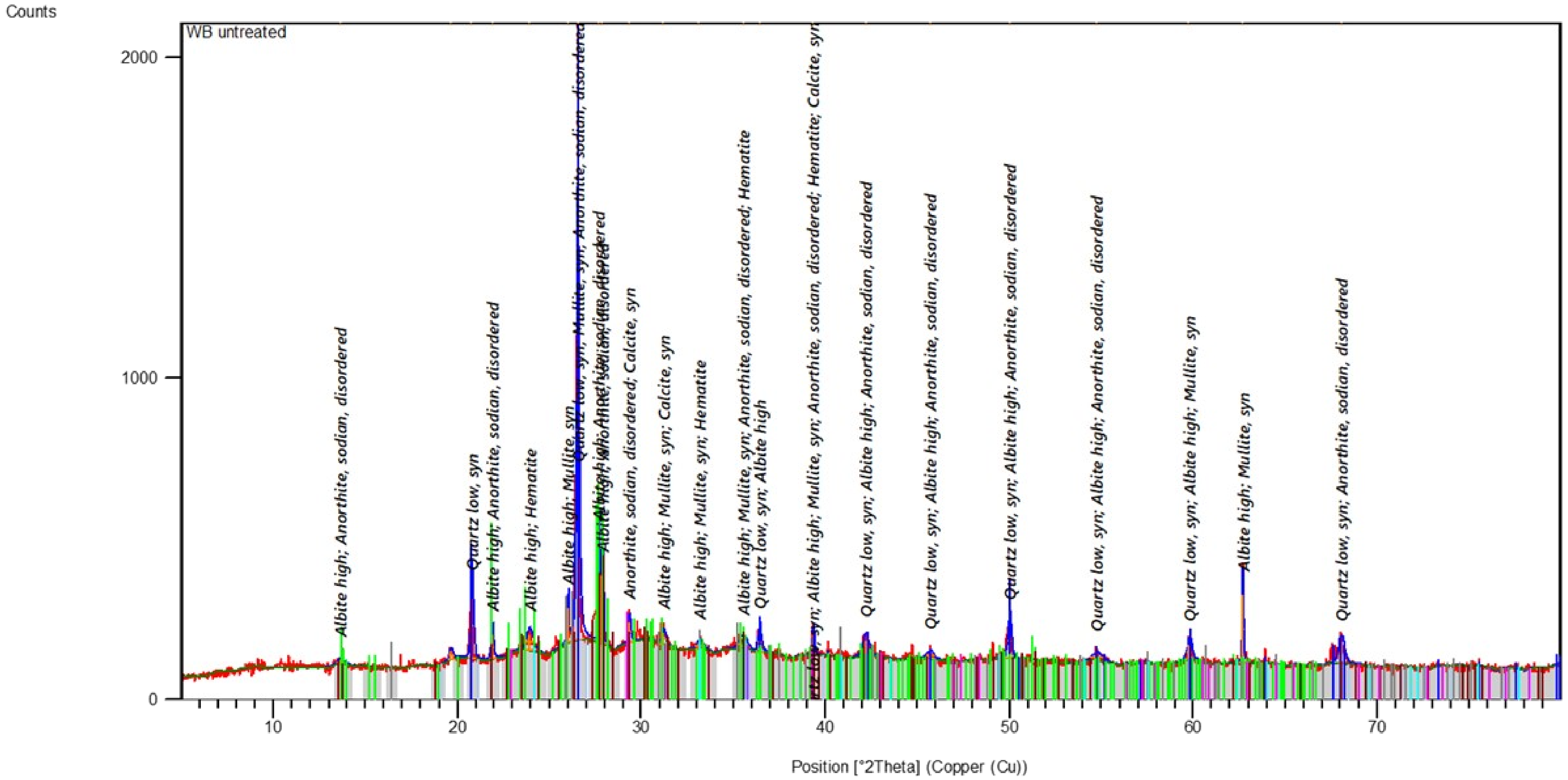

The XRD pattern of waste brick

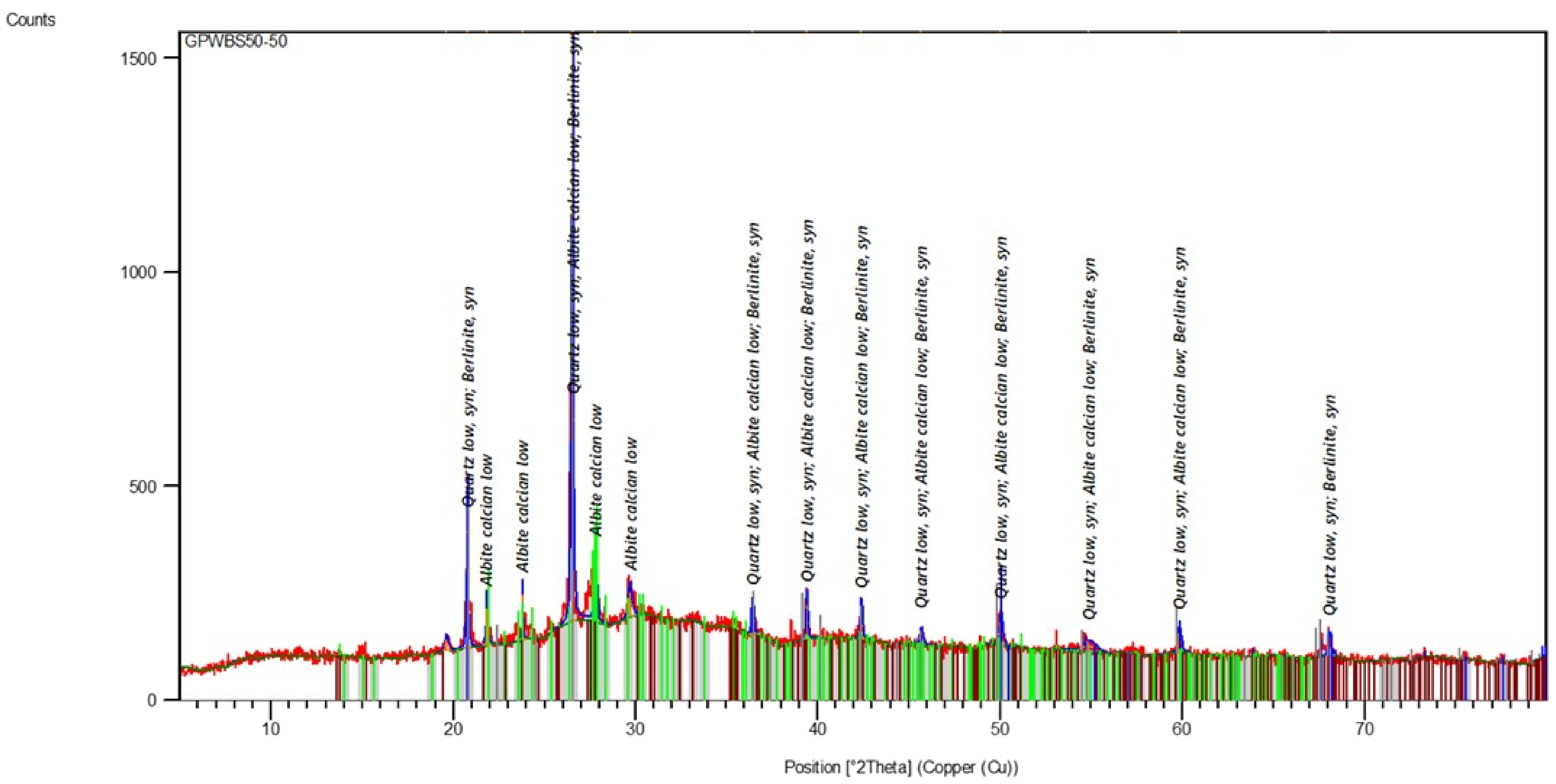

The XRD pattern of salt-activated foamed geopolymer.
